# Key benefits of dexamethasone and antibody treatment in COVID-19 hamster models revealed by single-cell transcriptomics

**DOI:** 10.1016/j.ymthe.2022.03.014

**Published:** 2022-03-24

**Authors:** Emanuel Wyler, Julia M. Adler, Kathrin Eschke, G. Teixeira Alves, Stefan Peidli, Fabian Pott, Julia Kazmierski, Laura Michalick, Olivia Kershaw, Judith Bushe, Sandro Andreotti, Peter Pennitz, Azza Abdelgawad, Dylan Postmus, Christine Goffinet, Jakob Kreye, S Momsen Reincke, Harald Prüss, Nils Blüthgen, Achim D. Gruber, Wolfgang M. Kuebler, Martin Witzenrath, Markus Landthaler, Geraldine Nouailles, Jakob Trimpert

**Affiliations:** 1 Berlin Institute for Medical Systems Biology (BIMSB), Max Delbrück Center for Molecular Medicine in the Helmholtz Association (MDC), Berlin, Germany; 2 Institute of Virology, Freie Universität Berlin, Berlin, Germany; 3 Charité - Universitätsmedizin Berlin, Corporate Member of Freie Universität Berlin and Humboldt-Universität zu Berlin, Institute of Pathology, Berlin, Germany; 4 IRI Life Sciences, Institute for Biology, Humboldt-Universität zu Berlin, Berlin, Germany; 5 Charité - Universitätsmedizin Berlin, Corporate Member of Freie Universität Berlin and Humboldt-Universität zu Berlin, Institute of Virology, Berlin, Germany; 6 Berlin Institute of Health (BIH), Berlin, Germany; 7 Charité - Universitätsmedizin Berlin, Corporate Member of Freie Universität Berlin and Humboldt-Universität zu Berlin, Institute of Physiology, Berlin, Germany; 8 Institute of Veterinary Pathology, Freie Universität Berlin, Berlin, Germany; 9 Bioinformatics Solution Center, Freie Universität Berlin, Berlin, Germany; 10 Charité - Universitätsmedizin Berlin, Corporate Member of Freie Universität Berlin and Humboldt-Universität zu Berlin, Division of Pulmonary Inflammation, Berlin, Germany; 11 Charité - Universitätsmedizin Berlin, Corporate Member of Freie Universität Berlin and Humboldt-Universität zu Berlin, Department of Infectious Diseases and Respiratory Medicine, Berlin, Germany; 12 German Center for Neurodegenerative Diseases (DZNE) Berlin, Helmholtz Innovation Lab BaoBab (Brain Antibody-Omics and B-Cell Lab), Berlin, Germany; 13 Charité - Universitätsmedizin Berlin, Corporate Member of Freie Universität Berlin and Humboldt-Universität zu Berlin, Department of Neurology and Experimental Neurology, Berlin, Germany; 14 Charité - Universitätsmedizin Berlin, Corporate Member of Freie Universität Berlin and Humboldt-Universität zu Berlin, Department of Pediatric Neurology, Berlin, Germany

**Keywords:** COVID-19 treatment, dexamethasone, antibody, monoclonal antibody therapy, hamster, scRNA-seq, transcriptomics, SARS-CoV-2

## Abstract

For coronavirus disease 2019 (COVID-19), effective and well-understood treatment options are still scarce. Since vaccine efficacy is challenged by novel variants, short-lasting immunity, and vaccine hesitancy, understanding and optimizing therapeutic options remains essential.

We aimed at better understanding the effects of two standard-of-care drugs, dexamethasone and anti-severe acute respiratory syndrome coronavirus 2 (SARS-CoV-2) antibodies, on infection and host responses. By using two COVID-19 hamster models, pulmonary immune responses were analyzed to characterize effects of single or combinatorial treatments.

Pulmonary viral burden was reduced by anti-SARS-CoV-2 antibody treatment and unaltered or increased by dexamethasone alone. Dexamethasone exhibited strong anti-inflammatory effects and prevented fulminant disease in a severe disease model. Combination therapy showed additive benefits with both anti-viral and anti-inflammatory potency. Bulk and single-cell transcriptomic analyses confirmed dampened inflammatory cell recruitment into lungs upon dexamethasone treatment and identified a specifically responsive subpopulation of neutrophils, thereby indicating a potential mechanism of action.

Our analyses confirm the anti-inflammatory properties of dexamethasone and suggest possible mechanisms, validate anti-viral effects of anti-SARS-CoV-2 antibody treatment, and reveal synergistic effects of a combination therapy, thus informing more effective COVID-19 therapies.

## Graphical abstract

**Figure:**
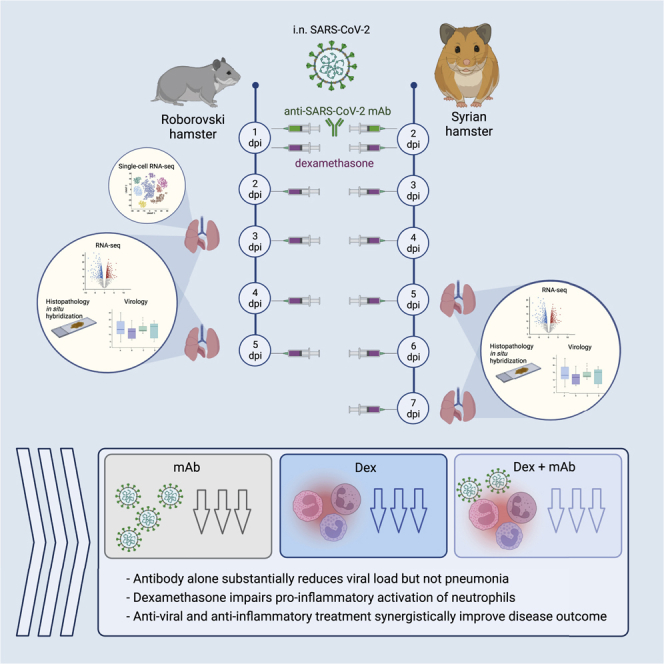

## Teaser

Wyler et al. explore dexamethasone and monoclonal antibody treatment of COVID-19 in two distinct hamster models. Using single-cell transcriptomics, they characterize treatment effects, ascertain mechanisms of therapeutic action, and underline the key role of neutrophils as drivers of and therapeutic targets in COVID-19.

## Introduction

A novel coronavirus (CoV), severe acute respiratory syndrome CoV-2 (SARS-CoV-2) emerged in December 2019 in Wuhan, China and evolved rapidly into an ongoing pandemic.^1^ While development of vaccines was successful, there is still a lack of approved, effective, and well-understood CoV disease 2019 (COVID-19) treatments.^2^^,^^3^

To devise successful host-directed therapeutic strategies, understanding of COVID-19 pathogenesis is required. For COVID-19 patients, virus-triggered exuberant cytokine release and associated tissue damage play a crucial role in disease severity, e.g., elevated levels of pro-inflammatory cytokines, as well as loss of effector T cells were associated with fatal outcomes.^4^, ^5^, ^6^, ^7^ Despite growing knowledge regarding the mechanisms of severe disease, very few treatment options are available, so that the use of corticosteroids, specifically dexamethasone, remains the treatment of choice for many critically ill patients.

Initially, use of corticosteroids was not recommended in treatment guidelines due to their broadly immunosuppressive action.^8^, ^9^, ^10^ Evidently, glucocorticoid treatment can result in impaired virus clearance.^11^ Nevertheless, in the RECOVERY trial, clinical application of dexamethasone yielded positive effects, especially for COVID-19 patients requiring oxygen therapy.^12^ Although corticosteroids are now used routinely to treat critically ill COVID-19 patients, putative hazards for mild to moderate COVID-19 patients as well as mechanisms underlying its protective efficacy in severe COVID-19 remain obscure and only begin to be investigated in greater depth.^13^

Since the development of small-molecule inhibitors of virus replication is difficult, passive immunization using monoclonal antibodies (mAbs) became an important approach to COVID-19 therapy relatively early in the pandemic. SARS-CoV-2 cell entry inhibition by mAb targeting the receptor-binding domain (RBD) of the spike protein revealed high effectivity.^14^ Various anti-SARS-CoV-2 antibodies have been developed and are currently tested in *in vivo* models or in clinical trials.^15^, ^16^, ^17^ The first approved anti-SARS-CoV-2 mAb was REGN-COV2 a combination of the mAbs casirivimab and imdevimab. Effectivity depends on timing of therapy, as application early in disease can prevent high-risk outpatient hospitalization.^18^ In fact, the TICO trial demonstrated that application of neutralizing mAbs, sotrovimab and BRII-196 plus BRII-198, in already hospitalized COVID-19 patients failed to improve their clinical outcomes.^19^ Yet early therapy or prophylaxis reduces virus titers in the respiratory tract and consequently the risk of severe disease progression.^20^^,^^21^ The therapeutic activity of mAbs depends critically on the presence of their binding sites in currently circulating virus variants.^22^ Dexamethasone, in contrast, acts non-specifically on the hosts’ immune response and is less likely to lose therapeutic power to new variants if induced immune responses remain similarly pathogenic. Dexamethasone and mAbs target distinct pathological aspects of COVID-19, namely broad inflammation and the causative pathogen, respectively. To date, detailed understanding of the mechanisms behind the action of these two standard treatments is still not fully developed and recent clinical trials missed to evaluate their synergistic potential. Hamsters are well established and widely used animal models for COVID-19^23^ that were used previously to examine effects of glucocorticoid^24^ and anti-viral and glucocorticoid combination treatment^25^ *in vivo*. In these studies, beneficial anti-inflammatory effects of glucocorticoid treatment became evident; at the same time, virus replication was rather enhanced by glucocorticoids. This outcome provides rationale for applying glucocorticoid treatment together with virus-neutralizing mAbs, which is conceptually similar to combinatorial dexamethasone plus remdesivir treatment mentioned in the NIH COVID-19 treatment guidelines.^26^ Still, there is the need for more thorough characterization of mechanisms underlying drug action, preferably in more than one model organism. To fill this knowledge gap, we examined the therapeutic effects of dexamethasone and monoclonal anti-SARS-CoV-2 antibody treatment as well as their potential as synergistic combinatorial therapy in hamster models of moderate and severe COVID-19 using single-cell and bulk transcriptome-based analyses.

## Results

### Purpose and study design

This study aims to compare two widely used COVID-19 treatments, dexamethasone and mAbs, as well as a combination thereof. To this end, we employed two COVID-19 hamster models, the Syrian and the Roborovski hamster, representing moderate and more severe COVID-19-like disease, respectively. Twenty-four individuals of both species were experimentally infected with 1 × 10^5^ plaque-forming units (pfu) of the ancestral SARS-CoV-2 variant B.1 (BetaCoV/Germany/BavPat1/2020) and divided into four groups of six animals each that received either mAb (30 mg/kg, single treatment), dexamethasone (2 mg/kg/day), mAb (30 mg/kg, single treatment) and dexamethasone (2 mg/kg/day), or mock treatment (PBS, daily). Since the course of disease varies considerably between both species, we choose to apply treatment at the onset of clinical signs for each species, which is 24 h for Roborovski or 48 h post-infection for Syrian hamsters. To further account for species-specific differences, we scheduled three animals per group for sampling at 3 and 5 days post-infection (dpi) in case of Roborovski hamsters or 5 and 7 dpi for Syrian hamsters. Due to early onset of severe disease, two mAb-treated and one mock-treated Roborovski hamster reached defined humane endpoints at day 2 post-infection and had to be terminated ahead of schedule. Clinical and virological parameters were determined for each animal in this study; furthermore, lungs of Roborovski hamsters taken at day 3 were subjected to single-cell RNA (scRNA) sequencing to determine transcriptional response to infection and treatment on a single-cell level.

### Dexamethasone treatment prevents severe disease, while monoclonal antibodies decrease viral burden

**Figure 1:**
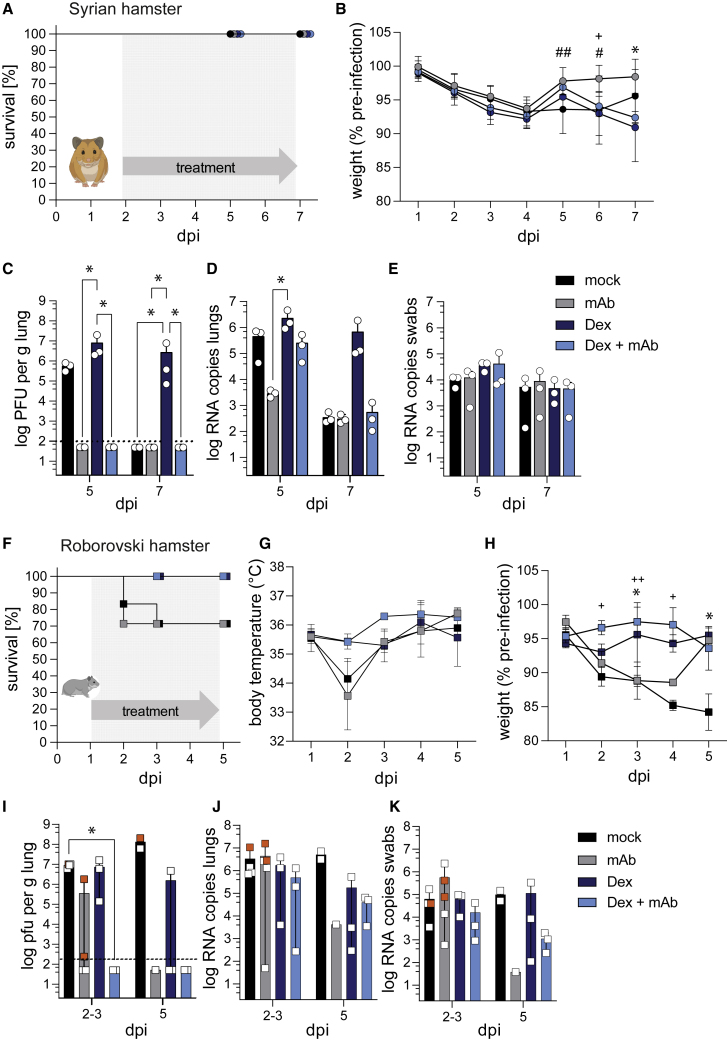
Clinics and virology of SARS-COV-2-infected Syrian and Roborovski hamsters under COVID-19 therapy (A–E) Syrian hamsters were challenged with SARS-CoV-2 (1 × 10^5^ pfu wild type [WT]) and treated once at 2 dpi with 30 mg/kg mAb CV07-209 (mAb; n = 6), daily starting at 2 dpi with 2 mg/kg dexamethasone (Dex; n = 6), or received combination treatment (Dex + mAb; n = 6). (A and B) Survival rates (A) in percent of SARS-CoV-2-infected Syrian hamsters and body weight (B) development in percent after virus challenge were measured until analysis time point (5 dpi, n = 3 and 7 dpi, n = 3) and displayed according to treatment group. (B) Results are displayed as mean ± SD. (C) Quantification of replication-competent virus as plaque-forming units (pfu) per gram homogenized lung tissue is shown. Dotted line marks the limit of detection (DL = 100 pfu). Titers below the detection limits were set to DL/2 = 50 pfu. (D and E) Number of genomic RNA (gRNA) copies detected in homogenized lung tissue (D) and oropharyngeal swabs (E) is shown. (C–E) Results are shown as mean with range. (F–K) Roborovski hamsters were challenged with SARS-CoV-2 (1 × 10^5^ pfu WT) and treated once at 1 dpi with 30 mg/kg mAb CV07-209 (mAb; n = 6), daily starting at 1 dpi with 2 mg/kg dexamethasone (Dex; n = 6), or received combination treatment (Dex + mAb; n = 6). (F–H) Survival rates (F) in percent of SARS-CoV-2-infected Roborovski hamsters, body temperature (G) in degree Celsius, and body weight (H) development in percent after virus challenge were measured until planned analysis time point (3 dpi and 5 dpi) or until termination due to score sheet criteria (non-survivors) according to treatment group. Two hamsters from the mAb group and one hamster from the mock-treated group were euthanized at 2 dpi (represented by orange squares; I–K). One hamster from the mock-treated group reached endpoint criteria at 3 dpi and was included in 3 dpi time point analysis as planned. (G and H) Results are displayed as mean ± SD. (I) Virus titers displayed as pfu per gram homogenized lung tissue are shown. Dotted line marks the limit of detection (DL = 100 pfu). Titers below the detection limits were set to DL/2 = 50 pfu. (J and K) Quantification of gRNA copies in homogenized lung tissue (J) and oropharyngeal swabs (K) is shown. (I–K) Results are displayed as mean with range. (A and F) Log rank test is shown. (B, G, and H) Two-way ANOVA is shown. Dunnett’s multiple comparisons test against mock is shown. ∗p < 0.05 (mock versus Dex); #p < 0.05 (mock versus mAb); ##p < 0.01 (mock versus mAb), +p < 0.05 (mock versus Dex + mAb), ++p < 0.01 (mock versus Dex + mAb). (C–E and I–K) Kruskal-Wallis is shown. Dunn’s multiple comparisons test is shown. ∗p < 0.05.

Following SARS-CoV-2 infection, Syrian hamsters lost body weight. Irrespective of treatment, Syrian hamsters failed to show significant differences in body weight development, nor did they present with severe signs of disease (Figures 1A and 1B). Titers of replication-competent virus of all hamsters receiving mAb or combination treatment were below the detectable level at all sampling time points. The use of dexamethasone alone increased viral titers in the lungs of Syrian hamsters and delayed viral clearance with moderately increased titers on day 5 and significantly increased titers at 7 dpi (Figure 1C). The same trend was also evident in virus genomic RNA (gRNA) levels in the lungs (Figure 1D), but not in the upper respiratory tract (Figure 1E), which is the common site of sampling in patients.

Contrary to Syrian hamsters, Roborovski hamsters, which can develop fulminant disease early after infection,^27^ displayed marked differences in clinical parameters in response to specific treatments. Specifically, both dexamethasone alone and in combination with mAb protected Roborovski hamsters from severe disease progression. By contrast, hamsters assigned to mAb treatment (2/6 on 2 dpi) and animals receiving mock treatment (2/6 on 2 dpi or 3 dpi) had to be euthanized prior to the terminal time point as they reached human endpoint criteria (Figure 1F). Hamsters that developed severe disease in respective groups presented with drastic drops in body temperature at 2 dpi (Figure 1G). Until the end of the experiment, body weights in the dexamethasone-treated groups remained stable, animals in the mAb treatment group recovered from initial weight losses, while mock-treated animals continued to lose weight throughout the experiment (Figure 1H). Similar to Syrian hamsters, replicating virus was below the detectable level in the lungs of Roborovski hamsters treated with either mAb or combinatorial therapy at days 3 and 5 post-infection. Only Roborovski hamsters that had to be terminated at 2 dpi showed high titers of replication-competent virus despite mAb treatment (Figure 1I). In contrast to the results obtained from Syrian hamsters, no boost of viral replication was observed in the dexamethasone-treated group of Roborovski hamsters compared with mock-treated animals. This result was evident for all time points on both replicating virus and virus gRNA level in the lungs as well as in the upper respiratory tract (Figures 1J and 1K).

### Dexamethasone restricts the inflammatory response

**Figure 2:**
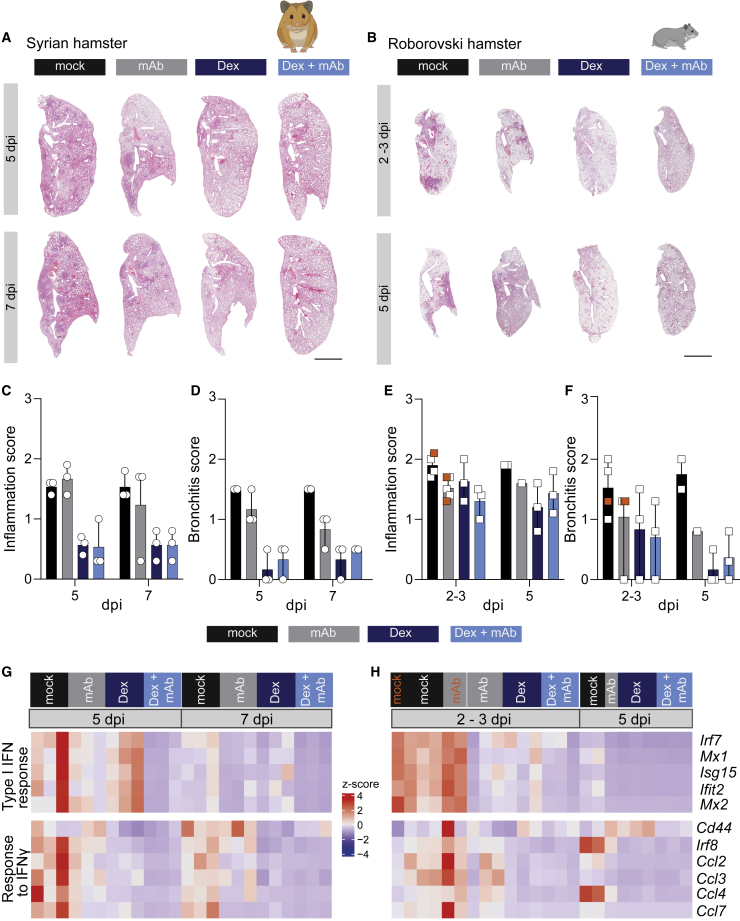
Dexamethasone treatment dampens inflammatory responses in SARS-CoV-2-infected hamsters (A and B) Longitudinal sections of H&E-stained left lungs from representative Syrian hamsters (A) and Roborovski hamsters (B) at indicated time points post-infection. Consolidated areas indicative of pneumonia appear in darker colors. Scale bars represent 3 mm. (C and E) Lung inflammation score (C, Syrian; E, Roborovski hamsters) accounting for the severities of pneumonia, immune cell influx, perivascular lymphocyte cuffs, bronchitis, bronchial epithelial necrosis, alveolar epithelial necrosis, and type II pneumocyte hyperplasia is shown. (D and F) Bronchitis score (D, Syrian; F, Roborovski hamsters) assessing bronchitis and bronchial epithelial necrosis is shown. (G and H) Gene expression (G, Syrian; H, Roborovski hamsters) was quantified using polyA RNA high-throughput sequencing from Syrian hamster lung samples. Shown are *Z* scores of fragments per kilo base of transcript per million mapped fragments (fpkm) values calculated over all samples on a color scale ranging from blue (−4) to red (+4) for selected genes. Time points and treatments are shown on top of the heatmap. Samples from animals euthanized at 2 dpi are shown in orange.

Dexamethasone is a useful drug to treat severe COVID-19 patients.^12^ To better characterize effects on local pathomechanisms, we performed lung histopathology upon dexamethasone, mAb, and combinatorial therapy against SARS-CoV-2 in models of moderate (Syrian hamster) and severe (Roborovski hamster) COVID-19 (Figures 2A–2F).

Lung histology indicated that, in both Syrian (Figure 2A) and Roborovski hamsters (Figure 2B), dexamethasone and combination treatment markedly reduced immune cell infiltrates over time (Figure S1). Inflammation and bronchitis scores were reduced from 5 dpi on in all groups receiving dexamethasone, which corresponds to 3 or 4 days post-treatment start for Syrian and Roborovski hamsters, respectively (Figures 2C–2F). mAb treatment alone reduced pneumonia, however, to a lesser extent as compared to dexamethasone (Figures 2A–2F and S1).

Next, we investigated how anti-viral and inflammatory transcriptional responses were influenced by treatment in Syrian (Figures 2G and S2A) and Roborovski hamsters (Figures 2H and S2B) over time. Therefore, we analyzed previously established viral-infection-related gene sets, *response to type I interferon* (*IFN*) and *IFN-gamma* (*IFN-γ*).^28^^,^^29^ In Syrian hamsters, the amplitude of the *type I IFN response* genes decreased from 5 to 7 dpi in the absence of treatment (Figures 2G and S2A). mAb treatment alone or in combination with dexamethasone led to further reduction in gene expression of the *type I IFN response* genes. In contrast, *IFN-γ response* set genes decreased more upon dexamethasone compared with mAb treatment (Figures 2G and S2A). Similar effects were observed in Roborovski hamsters (Figures 2H and S2B). The combination treatment led to a strong reduction of both gene sets, independent of hamster species (Figures 2G and 2H).

Taken together, treatment-related improvement in clinical parameters and histopathology correlated with substantially altered gene expression profiles in general and a reduced expression of the *response to IFN-γ* gene set following dexamethasone treatment specifically.

### Dexamethasone reduces influx of immune cells and stabilizes endothelial cells

As described above, both mAb and dexamethasone treatment, and in particular their combination, attenuated inflammatory aspects of pneumonia following SARS-CoV-2 infection, thereby mitigating the otherwise severe disease observed in Roborovski hamsters.

**Figure 3:**
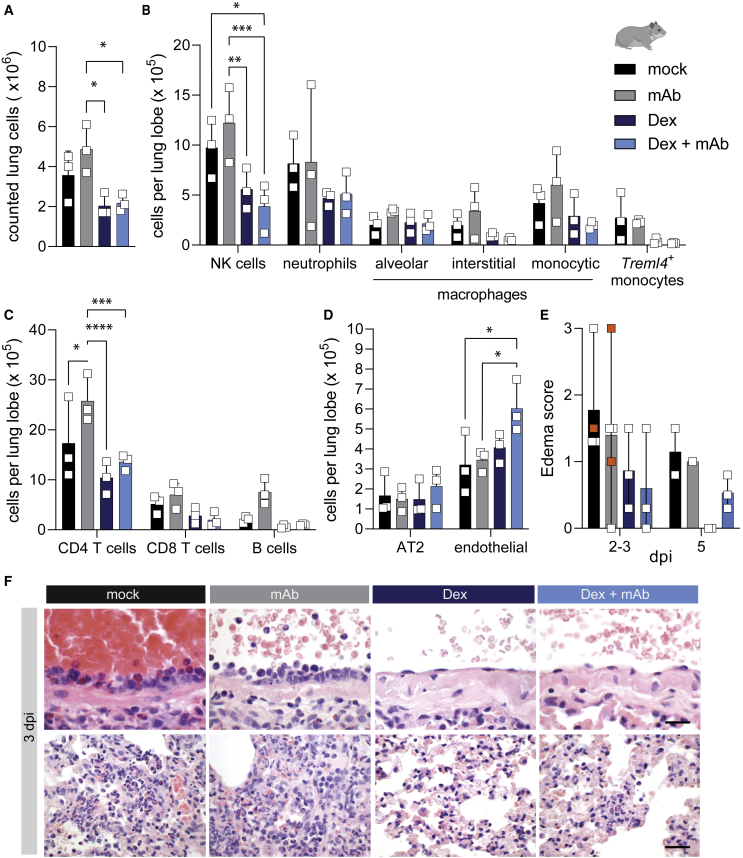
Dexamethasone limits immune cell recruitment in Roborovski hamsters Roborovski hamsters were challenged with SARS-CoV-2 (1 × 10^5^ pfu WT), treated once at 1 dpi with 30 mg/kg mAb CV07-209 (mAb), daily starting at 1 dpi with 2 mg/kg dexamethasone (Dex), or received combination treatment (Dex + mAb). At 3 dpi, n = 3 Roborovski hamsters of each group were subjected to pulmonary single-cell RNA sequencing analysis. Pulmonary single-cell suspensions were generated, cells microscopically counted, and total numbers per lung lobe calculated. (A) Cell count of isolated cells per lung lobe according to treatment group is shown. (B–D) Calculated numbers of indicated innate immune cells (B), T and B lymphocytes (C), and AT2 and endothelial cells (D) based on scRNA-seq-determined cell frequencies (Figure S3) and according to treatment group are shown. Data display means ± SD. n = 3 per group. (A–D) Two-way ANOVA and Tukey’s multiple comparisons test are shown. ∗p < 0.05, ∗∗p < 0.01, ∗∗∗p < 0.001, and ∗∗∗∗p < 0.0001. (E) Edema score resulting from semi-quantitative assessment of alveolar and perivascular edema is shown. (F) H&E-stained histopathology of pulmonary vascular endothelia (upper panel) and lung parenchyma (lower panel) from Roborovski hamsters at 3 dpi is shown. Mock- and mAb-treated groups had moderate to marked endothelialitis with activation and loss of endothelial cells, whereas the vascular endothelium remained mostly intact in Dex- and Dex + mAb-treated groups. The inflammatory response was more pronounced in mock- and mAb-treated hamsters compared with Dex- and Dex + mAb-treated animals. Differences were particularly observed for infiltrating neutrophils, macrophages, and lymphocytes as well as for the degree of alveolar epithelial cell necrosis. Scale bars: 15 μm (top) and 25 μm (bottom).

In order to investigate cellular mechanisms underlying these treatment effects, we next performed pulmonary scRNA sequencing (scRNA-seq) of Roborovski hamsters for all treatment groups at 3 dpi. First, we evaluated the absolute content and composition of cell types by measuring total cell counts of the dissociated tissue (Figure 3A) and relative cell type distribution from scRNA-seq data (Figures 3B–3D and S3A–S3J). Lungs from dexamethasone (alone or in combination with mAb)-treated hamsters yielded significantly lower total cell counts (Figure 3A). This reduction likely originated from reduced infection-triggered pulmonary immune cell immigration. NK cell numbers were significantly lower in dexamethasone-treated groups compared with mock- and mAb-treated hamsters; similarly, neutrophil, monocytic macrophage, *Treml4*^*+*^ monocyte, and T and B cell showed reduced numbers in hamsters receiving dexamethasone, although the difference was not statistically significant (Figures 3B and 3C). Notably, endothelial cells had significantly higher counts in groups treated with a combination therapy of dexamethasone and mAb (Figure 3D) as compared with mock-treated animals. Higher endothelial cell counts were likely caused by mechanisms governing endothelial protection, rather than cell proliferation, since increased *Mki67* and *Top2* expression was not detectable in endothelial cells (Figure S3K). The notion of endothelial protection was supported by histopathological analyses showing reduced edema formation and reduced endothelialitis in dexamethasone-treated groups (Figures 3E and 3F, upper panel), thus replicating findings in patients.^30^ However, this conclusion is limited by a lack of information on how treatment would have affected baseline endothelial cell numbers in naive animals. Histopathological analyses likewise confirmed reduction of recruited immune cells following single dexamethasone treatment alone and in combination with mAb (Figure 3F). In contrast to mAb treatment alone, dexamethasone therefore largely reduced recruitment of immune cells.

### Neutrophils and monocytic macrophages exhibit strong responses to dexamethasone

Dexamethasone directly impairs transcription of nuclear factor κB (NF-κB) target genes via Rela/p65 and Crebbp/CBP.^31^ In order to assess the effect of dexamethasone treatment, known target genes of the glucocorticoid receptor, the *coagulation cascade factor F13a1*,^32^ the plasma apolipoprotein *serum amyloid a-3 protein* (*Saa3*),^33^ and *Dusp1*/MKP-1, an inhibitor of the mitogen-activated protein (MAP) kinase pathways,^34^ were investigated (Figures S4A–S4C). Neutrophils and macrophages, particularly monocytic macrophages, from dexamethasone-treated groups showed strong increase in target gene expression, *F13a1*, *Dusp1*, and *Saa3* (Figures S4A–S4C).

**Figure 4:**
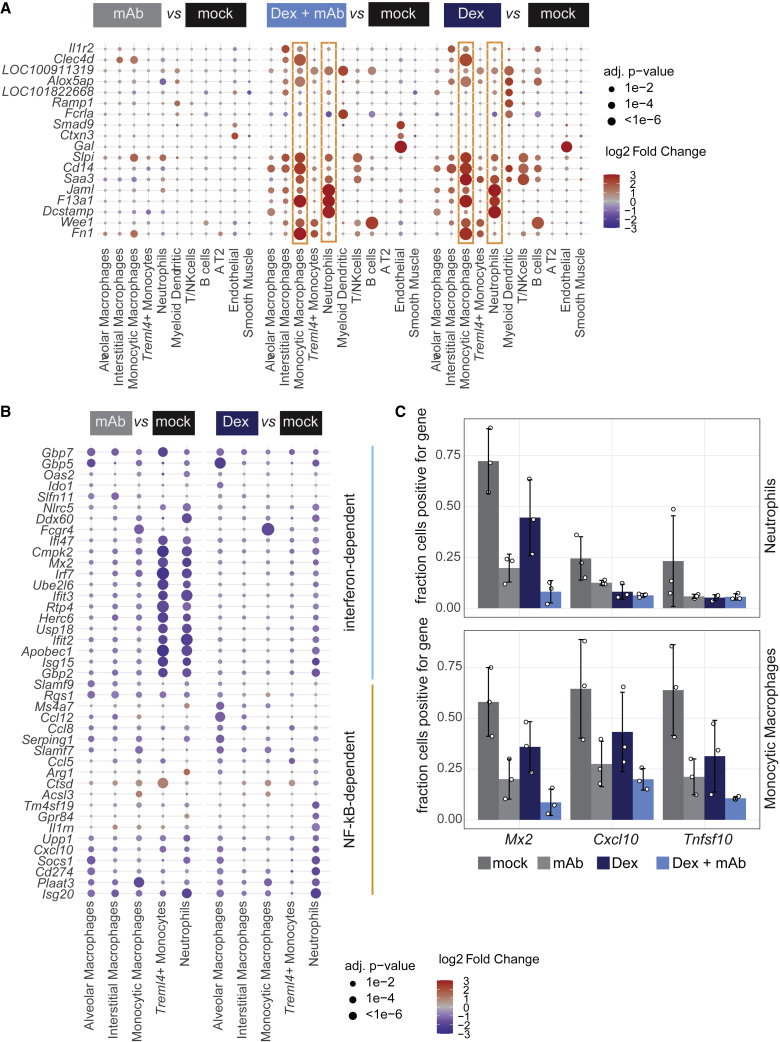
Macrophages and neutrophils show strongest gene expression changes following dexamethasone treatment (A) Shown are genes with at least 4-fold upregulation in at least one cell type in dexamethasone- compared with mock-treated animals, all three treatments are separately compared to mock-treatment. Size and colors of the dots indicate log2-transformed fold changes (FCs) and p values, respectively. Adjusted (adj) p values were calculated by DEseq2 using Benjamini-Hochberg corrections of two-sided Wald test p values. Genes are ordered by unsupervised clustering. (B) Shown are interferon- and NF-κB-dependent genes as determined in Figure S4 for the comparisons Dex versus mock and Dex + mAb versus mock. (C) Expression of *Mx2*, *Tnfsf10*, and *Cxcl10* in neutrophils (top) and monocytic macrophages (bottom). Shown are the fraction of cells with greater than or equal to one mRNA count (means ± SD; n = 3 per group).

For an unbiased view of the data, we selected all genes that were at least 4-fold upregulated in all cell types (Figure 4A). Again, monocytic macrophages and neutrophils stood out with several upregulated genes, including *Saa3* and *F13a1*, as mentioned above. We identified a dexamethasone-induced transcriptional program common to several cell types, whereas some genes, for example, *Gal* (coding for galanin and galanin message-associated peptides) in endothelial cells were cell type specific. In contrast, tissue cells, including endothelial cells, alveolar epithelial cell type 2 (AT2), or smooth muscle cells, did not show substantial upregulation of gene expression in response to dexamethasone alone (Figure 4A). Notably, the mRNA of the glucocorticoid receptor, encoded by the *Nr3c1* gene, is ubiquitously present in both Roborovski hamsters and Syrian hamsters, and not modulated by SARS-CoV-2 infection or the employed treatments (Figure S4D).

Next, we asked which disease-relevant changes in gene expression were influenced by treatment in different cell types. We therefore assessed changes in gene expression between treatments for each cell type in an unbiased manner (Figure S4E). We noticed consistent downregulation of a group of IFN-induced genes (IFN-stimulated genes [ISGs]), such as *Ifit2/3*, *Ifi27*, and *Ifi209* in animals treated with mAb alone or in combination with dexamethasone, but not with dexamethasone alone. Conversely, some genes, such as *Tnfsf10* (coding for the pro-inflammatory cytokine Trail) in neutrophils, were more reduced in dexamethasone-treated compared with mAb-treated animals.

In order to understand the changes in gene expression patterns caused by these treatments, we defined, based on our Syrian hamster scRNA-seq data,^28^ two groups of gene sets. The first was viral pathogen-associated molecular pattern (PAMP) dependent (identified as “NF-κB dependent”), the second induced by the infection in general (“IFN dependent”; Figure S4F). Whereas the IFN-dependent gene expression was reduced more by mAb compared with dexamethasone treatment, for the “NF-κB-dependent” gene set, we in tendency observed the opposite (Figure 4B). We scrutinized this effect in detail in monocytic macrophages and neutrophils and found that, in neutrophils, the downregulation of the NF-κB-driven cytokine genes Cxcl10 and Tnfsf10 in tendency experience stronger downregulation by dexamethasone compared with the ISG Mx2 (Figure 4C). For all genes, the combination treatment showed an additive effect (Figure 4).

Overall, these data suggest that the reduced viral load in mAb-treated animals leads to a generally reduced anti-viral/type 1 IFN signal, whereas dexamethasone treatment downregulates specific genes in some cell types, such as the pro-inflammatory cytokines *Tnfsf10* and *Cxcl10* in neutrophils, thereby attenuating classic features of pneumonia in animals receiving dexamethasone.

### Dexamethasone alters the neutrophilic response to SARS-CoV-2 infection

**Figure 5:**
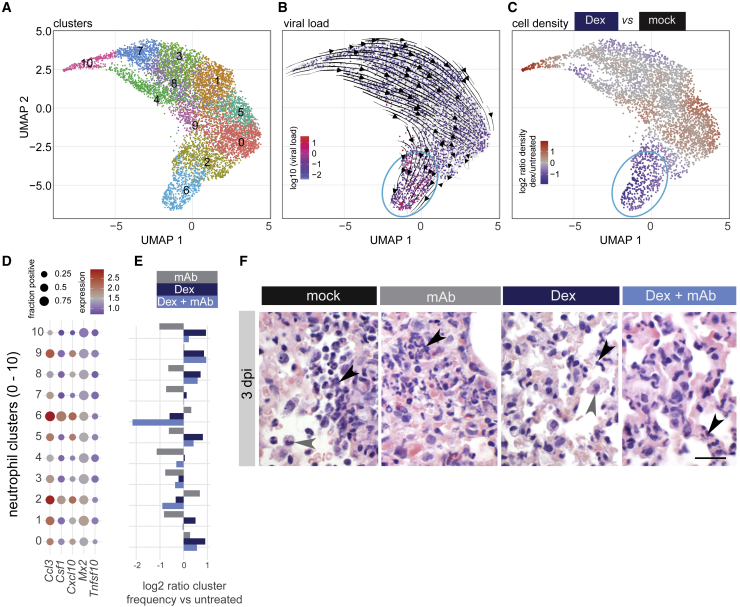
Absence of a specific chemokine-expressing subset of neutrophils upon dexamethasone treatment in Roborovski hamsters (A) Neutrophils from the scRNA-seq data were sub-clustered using the Louvain algorithm based on their individual transcriptomes and two-dimensional projections performed using the uniform manifold approximation and projection (UMAP) algorithm. Cells were colored by their cluster identity. (B) Projection is as in (A), but cells are colored by the log10-transformed percentage of viral RNA. Overlaid are the stream arrows derived from an RNA velocity analysis. Neutrophil cluster 6 is marked with a light blue oval. (C) Changes in cellular density on the UMAP projection were calculated and cells colored by fold changes of the indicated Dex versus mock. Red indicates increased density, and blue indicates decreased density. Neutrophil cluster 6 is marked with a light blue oval. (D) Dot plots show the expression of selected genes over all hamsters in the clusters as defined in (A). The dot size indicates the fraction of cells in the clusters as indicated on the left from mock-treated animals, with greater than or equal to one mRNA count for the respective gene. Color represents average expression in those cells. (E) Graph indicates the log2-transformed fold changes of the cell counts in the respective neutrophil clusters 1–10, with all three treatments compared with mock. For example, in cluster 6, there are about one-third less cells (dark blue bar at −0.6, which corresponds to log2 of 0.66) upon dexamethasone treatment. (F) Histopathology of Roborovski hamsters 3 days after infection revealed moderate to marked alveolar and interstitial infiltration with viable and degenerate neutrophils (black arrowheads) in mock- and mAb-treated animals as well as elevated numbers of alveolar macrophages (gray arrowhead). Dex- and Dex + mAb-treated hamsters had lower numbers of neutrophils, especially in their alveolar spaces and mild to moderate numbers of neutrophils in alveolar capillaries (black arrowheads). Activated alveolar macrophages phagocytized cellular debris and cleared the inflammatory response (gray arrowhead). Scale bar represents 20 μm.

Given that neutrophils are critical drivers of immune pathology and showed a particularly strong reactivity to dexamethasone treatment, we investigated this cell type in greater detail. For this, we sub-clustered the neutrophil population into 11 subpopulations (Figure 5A).

In order to understand the transcriptional dynamics within neutrophils and the influence of the treatments used here, we performed an RNA velocity analysis that can predict the future state of individual cells.^35^^,^^36^ This showed a transcriptional trend toward the cluster on the bottom of the projection (cluster 6 in Figure 5A), which also showed a particularly high viral RNA content (Figures 5B and S5A). Importantly, cell density in that cluster decreased upon dexamethasone treatment (Figures 5C and S5B).

Among the genes that were particularly prominent in cluster 6 were the cytokines and macrophage and lymphocyte attractants *Csf1* and *Ccl3* (Figure S5C).^37^^,^^38^ We therefore plotted the expression of these two genes along with the ISG/NF-κB targets *Mx2/Tnfsf10/Cxcl10*, which showed that neutrophils in cluster 6 express *Csf1* and *Ccl3* at particularly high levels (Figure 5D); at the same time, these cells become less abundant upon dexamethasone and particularly combination treatment (Figure 5E). Concomitantly, by histopathology analysis, we observed less neutrophils in the dexamethasone-treated groups (Figure 5F). Of note, cells expressing mRNAs of receptors (*Csf1r*, *Ccr1*, *Ccr4*, and *Ccr5*) corresponding to cytokines *Csf1* and *Ccl3* were less abundant in the lungs upon dexamethasone treatment (Figure S5D; compare with Figures 3B and S3B). In addition, neutrophil-cluster 6 showed particularly low and high expression of *Il1r2* and *Isg20* (Figure S5E), respectively, thereby recapitulating the phenotypes seen for immunosuppressive and IFN^active^ neutrophils in the peripheral blood of COVID-19 patients.^13^

To generalize the observation of this transcriptional dynamic, we applied diffusion map analysis of neutrophils to identify their most prominent direction of variation (Figure S5F).^39^^,^^40^ For each treatment, we show the neutrophil density along the diffusion axis (Figure S5G, upper part), which we defined as the first non-trivial component of the diffusion map. The directional progression toward the right on this axis (which is the same cellular state represented as neutrophil-cluster 6 above) is present in all conditions, as shown by the average RNA velocity projected onto the diffusion axis (Figure S5G, lower part). However, most neutrophils derived from hamsters treated with dexamethasone or combinatorial treatment are found at the leftmost part of the axis, whereas neutrophils from hamsters with mAb and mock treatment are split into a left and right part, confirming that, with dexamethasone treatment, an otherwise directional progression of neutrophils is limited. In order to relate the diffusion axis to biological effects, we scored hallmark signatures^41^ for every neutrophil and linearly correlated each hallmark with the diffusion axis (Figure S5H, upper part). In addition, we correlated the expression profiles of each gene with the diffusion axis (Figure S5H, lower part). These correlations revealed that the drive toward neutrophil-cluster 6 marked by high expression of *Csf1* and *Ccl3* and elevated amounts of viral RNA is accompanied by an increase of interferon and inflammatory response gene expression (such as *Isg15* or *Cd274*) and a decrease in the levels of classical neutrophil marker genes, such as *S100a8/9* or *Pglyrp1*. Dexamethasone limits this dynamic, effectively keeping the neutrophils in a stationary transcriptomic state at the left part of the diffusion axis. As we will discuss in detail, this stagnation could be a reason for the reduced production of lymphocyte attractants and, consequently, the reduction of lung infiltrates.

## Discussion

In this study, we examined the effects of separate and combined anti-viral and anti-inflammatory treatments for COVID-19 in two hamster models reflecting a moderate (Syrian hamster) and severe (Roborovski hamster) disease course, respectively. Using histopathology and bulk and single-cell transcriptomic analysis of hamsters subjected to dexamethasone, mAb, and combination treatment, we demonstrate treatment efficacy and identified a subset of neutrophils that express macrophage- and lymphocyte-attracting cytokines and can be impeded by dexamethasone.

The use of dexamethasone caused a boost of virus replication and a significant delay of viral clearance in Syrian hamsters, albeit without significantly worsening the clinical course of disease. In the light of existing literature on the enhanced replication of respiratory viruses upon dexamethasone treatment^42^ and data that overall show a tendency toward a boost of SARS-CoV-2 replication in dexamethasone-treated patients,^11^^,^^43^, ^44^, ^45^ this result is not unexpected and may imply a risk for increased and/or prolonged transmissibility. Still, dexamethasone exerted the expected anti-inflammatory effects and attenuated inflammatory lung injury. As previously reported,^16^ the mAb CV07-209 employed in this study effectively abolished virus replication within 48 h of treatment. At the dose applied here, the mAb inhibited the boost of virus replication after dexamethasone treatment. This suggests that a combination of dexamethasone and mAb may present an effective way to reduce inflammation and at the same time suppress virus replication, limiting the risk of viral transmission. This would advocate for the use of a combination therapy in patients at risk of severe disease relatively early when active virus replication is still ongoing and before lung injury or COVID-19-triggered fibrosis^46^ develop. Post hoc analysis of clinical trials investigating the efficacy of neutralizing mAbs with a focus on patient subsets that had received dexamethasone as standard of care could aid in evaluating the clinical suitability of such a combination. Interestingly, the use of dexamethasone in the Roborovski hamster, a species highly susceptible to severe COVID-19-like disease, did not boost virus replication at any of the examined time points. One possible explanation could be that the virus-restrictive immunity targeted by dexamethasone in Syrian hamsters is dysregulated in Roborovski hamsters, and consequently, its inhibition has no impact on viral control.

Treatment of SARS-CoV-2-infected hamsters with dexamethasone reduced the extent of lung infiltrates, comparable to what can be observed in computed tomography (CT) scans of human COVID-19 patients.^47^ In the scRNA-seq analysis, this effect was evident as reduced abundance of infiltrating leukocytes and lymphocytes. In an unbiased comparison of gene expression patterns in the different lung cell types, we found that neutrophils are particularly affected by dexamethasone treatment. A detailed analysis showed that, upon SARS-CoV-2 infection, neutrophils move toward a state with high expression of the cytokines *Csf1* and *Ccl3* and that this movement is impaired by dexamethasone. Furthermore, the receptors of the two cytokines are expressed on a range of cell types that become less abundant in the lungs upon dexamethasone treatment. This together suggests a mechanistic link underlying the protective effect, through reduction of lung infiltrates, by dexamethasone. These results are in line with the key role of neutrophils in COVID-19 pathogenesis^48^ and corroborate recent findings highlighting the effect of dexamethasone on neutrophils in peripheral blood.^13^ Although neutrophils in blood and lung might not be directly comparable, the observation by Sinha and colleagues, a neutrophil “IFNactive” program restrained by dexamethasone, was similarly observed in the present study.

In addition to its effects on polymorphonuclear leukocytes (PMNs), dexamethasone treatment exerted protective effects on the endothelium of SARS-CoV-2-infected hamsters, likely by reducing endothelial injury caused by cytotoxic immunity and bystander effects conveyed by the pro-inflammatory program executed by highly stimulated immune cells. As a secondary effect, the expression of inflammatory mediators by endothelial cells could also be reduced. Of clinical relevance, endothelial protection will reduce the development of lung edema and micro-thrombosis and may thus contribute to improved gas exchange in dexamethasone-treated patients.

Care should be taken not to transfer findings from animals uncritically to patients. Yet it should be noted that we and others recently demonstrated comparability between immunological responses and pulmonary phenotypes in hamsters and humans in response to SARS-CoV-2 infection.^28^^,^^49^^,^^50^ That notwithstanding, future studies should ideally compare patient data with the findings reported here with the obvious constraint of limitations in the availability of corresponding human biomaterial.

In summary, we found that broadly active anti-inflammatory and immunosuppressive agents, such as dexamethasone, may have a strong benefit in SARS-CoV-2 infection at high risk for severe disease when applied before the onset of severe illness, particularly when combined with an anti-viral agent. A recent analysis showed that COVID-19-related acute respiratory distress syndrome (ARDS) patients can be classified into hypo- and hyperinflammatory types, with corticosteroid treatment being beneficial only for the latter.^51^ Animal models as the ones described here can help to better dissect causes and types of COVID-19 lung pathologies and thus help to improve therapeutic strategies.

## Materials and methods

An online supplement is provided, giving more details on the methods described here.

### Ethics statement and COVID-19 hamster models

Experiments including female and male Syrian hamsters (*Mesocricetus auratus*; breed RjHan:AURA, JanvierLabs, France) and Roborovski hamsters (*Phodopus roborovskii*, obtained via the German pet trade) were approved and executed in compliance with all applicable regulations (Landesamt für Gesundheit und Soziales Berlin, permit number 0086/20). SARS-CoV-2 (BetaCoV/Germany/BavPat1/2020) preparation^52^ and intranasal infection of hamsters with 1 × 10^5^ pfu were carried out as previously described.^27^^,^^53^ Treatments were applied as single intraperitoneal (i.p.) treatment with 30 mg/kg mAb CV07-209 previously described to be effective against the ancestral B.1 SARS-CoV-2 variant used in this study^16^ and daily intramuscular (i.m.) treatment with 2 mg/kg dexamethasone in the respective groups. Hamsters were monitored daily until they reached scheduled take-out time points or defined humane endpoints. Virus titers and RNA copies were determined by plaque assay and quantitative RT-PCR analysis as previously described.^53^

### Histopathology and *in situ* hybridization of SARS-CoV-2 RNA

For histopathology and *in situ* hybridization (ISH), lungs were processed and tissues evaluated by board-certified veterinary pathologists in a blinded fashion following standardized recommendations, including pneumonia-specific scoring parameters as described previously.^54^

### Annotations of the *M. auratus* and *P. roborovskii* genome

The *M. auratus* genome was derived from Ensembl and modified as previously described.^28^ The detailed description of the *de novo* gene assembly of the Roborovski hamster genome was deposited on a pre-print server.^55^

### Bulk RNA analysis

For RNA bulk sequencing of both hamster species, the right medial lung lobe was removed and RNA isolated using Trizol reagent according to the manufacturer’s instructions. Bulk RNA sequencing libraries were constructed using the Nebnext Ultra II Directional RNA Library Prep Kit (New England Biolabs) and sequenced on a Nextseq 500 or Novaseq 6000 device. Reads were aligned to the genome using hisat2^56^ and gene expression quantified using quasR.^56^

### Single-cell RNA sequencing

To enable scRNA-seq, cells were isolated from Roborovski hamsters’ caudal lung lobe as previously described.^28^ One million lung cells per sample were subjected to cell multiplexing oligo (CMO) labeling according to manufacturers’ instructions (3ʹ CellPlex Kit Set A; 10× Genomics). Labeled cells from 12 samples were pooled, filtered, and counted. Pooled cells were adjusted to a final concentration of ∼1,600 cells/μL, and 197,760 cells were split into four equal pools and subjected to partitioning into Gel-Beads-in-Emulsions with the aim of recovering a maximum of 120,000 single cells from four lanes by following the instructions of Chromium Next GEM Single Cell 3ʹ Reagent Kits v.3.1 (Dual Index) provided by the manufacturer (10× Genomics). Library sequencing was performed on a Novaseq 6000 device (Illumina), with SP4 flow cells (read1: 28 nt; read2: 150 nt). Sequencing of one of four libraries failed.

### Analysis of single-cell RNA sequencing data

Analysis of the single-cell data was based on Seurat.^57^ Raw and processed data are available through GEO at GEO: GSE191080, code through Github at GitHub: Berlin-Hamster-Single-Cell-Consortium/Dwarf-Hamster-Dexamethasone-Antibody. Details on single-cell analysis and RNA velocity analysis can be found in the online supplemental information.

## Data and code availability

Raw and processed data are available through GEO at GEO: GSE191080 , code through Github at GitHub: Berlin-Hamster-Single-Cell-Consortium/Dwarf-Hamster-Dexamethasone-Antibody.

## Supplemental information

Document S1. Supplemental methods and Figures S1–S5

Document S2. Article plus supplemental information
